# Association Between Lifestyle Factors, Vitamin and Garlic Supplementation, and Gastric Cancer Outcomes: A Secondary Analysis of a Randomized Clinical Trial

**DOI:** 10.1001/jamanetworkopen.2020.6628

**Published:** 2020-06-26

**Authors:** Yang Guo, Zhe-Xuan Li, Jing-Yu Zhang, Jun-Ling Ma, Lian Zhang, Yang Zhang, Tong Zhou, Wei-Dong Liu, Zhong-Xiang Han, Wen-Qing Li, Kai-Feng Pan, Wei-Cheng You

**Affiliations:** 1 Key Laboratory of Carcinogenesis and Translational Research (Ministry of Education–Beijing), Department of Cancer Epidemiology, Peking University Cancer Hospital and Institute, Beijing, China; 2 Linqu County Public Health Bureau, Shandong, China

## Abstract

**Importance:**

The associations of lifestyle factors with gastric cancer (GC) are still underexplored in populations in China. Long-term nutritional supplementation may prevent GC in high-risk populations, but the possible effect modification by lifestyle factors remains unknown.

**Objective:**

To evaluate how lifestyle factors, including smoking, alcohol intake, and diet, may change the risk of GC incidence and mortality and whether the effects of vitamin and garlic supplementation on GC are associated with major lifestyle factors.

**Design, Setting, and Participants:**

This is a secondary analysis of the Shandong Intervention Trial, a masked, randomized, placebo-controlled trial that aimed to assess the effect of vitamin and garlic supplementations and *Helicobacter pylori* treatment on GC in a factorial design with 22.3 years of follow-up. The study took place in Linqu County, Shandong province, China, a high-risk area for GC. Data were collected from Jully 1995 to December 2017. Overall, 3365 participants aged 35 to 64 years identified in 13 randomly selected villages who agreed to undergo gastroscopy were invited to participate in the trial and were included in the analysis. Data analysis was conducted from March to May 2019.

**Interventions:**

Participants received vitamin and garlic supplementation for 7.3 years, *H pylori* treatment for 2 weeks (among participants with *H pylori* ), or placebo.

**Main Outcomes and Measures:**

The primary outcomes were GC incidence and GC mortality (1995-2017). We also examined the progression of gastric lesions (1995-2003) as a secondary outcome.

**Results:**

Of the 3365 participants (mean [SD] age, 47.1 [9.2] years; 1639 [48.7%] women), 1677 (49.8%) were randomized to receive active vitamin supplementation, with 1688 (50.2%) receiving placebo, and 1678 (49.9%) receiving active garlic supplementation, with 1687 (50.1%) receiving placebo. Overall, 151 GC cases (4.5%) and 94 GC deaths (2.8%) were identified. Smoking was associated with increased risk of GC incidence (odds ratio, 1.72; 95% CI, 1.003-2.93) and mortality (hazard ratio [HR], 2.01; 95% CI, 1.01-3.98). Smoking was not associated with changes to the effects of vitamin or garlic supplementation. The protective effect on GC mortality associated with garlic supplementation was observed only among those not drinking alcohol (never drank alcohol: HR, 0.33; 95% CI, 0.15-0.75; ever drank alcohol: HR, 0.92; 95% CI, 0.55-1.54; *P* for interaction = .03), and significant interactions were only seen among participants with *H pylori *(never drank alcohol: HR, 0.31; 95% CI, 0.12-0.78; ever drank alcohol: HR, 0.91; 95% CI, 0.52-1.60; *P *for interaction = .04). No significant interactions between vitamin supplementation and lifestyle factors were found.

**Conclusions and Relevance:**

In this secondary analysis of a randomized clinical trial, smoking was associated with an increased risk of GC incidence and mortality. Not drinking alcohol was associated with a stronger beneficial effect of garlic supplementation on GC prevention. Our findings provide new insights into lifestyle intervention for GC prevention, suggesting that mass GC prevention strategies may need to be tailored to specific population subgroups to maximize the potential beneficial effect.

**Trial Registration:**

ClinicalTrials.gov Identifier: NCT00339768

## Key Points

**Question:**

Are lifestyle factors associated with increased risk of gastric cancer (GC), and are they associated with changes in the long-term effects of vitamin and garlic supplementation on GC prevention in high-risk populations in China?

**Findings:**

In this secondary analysis of a randomized clinical trial with 3365 participants, smoking, but not alcohol intake, was associated with increased risk of GC incidence and mortality; the beneficial effect of garlic supplementation on GC prevention was stronger for individuals who did not drink alcohol.

**Meaning:**

The findings of this study provide evidence on the association of lifestyle factors with GC in high-risk populations and suggest that mass GC prevention strategies should be tailored to specific population subgroups to maximize potential beneficial effects.

## Teaser

This secondary analysis of a randomized clinical trial evaluates how lifestyle factors, including smoking, alcohol intake, and diet, may change the risk of gastric cancer (GC) incidence and mortality and whether the effects of vitamin and garlic supplementation on GC are associated with major lifestyle factors.

## Introduction

Gastric cancer (GC) is the third leading cause of cancer death worldwide.^1^ Linqu County, a rural area in Shandong province in northeastern China, has among the highest GC mortality rates in the world.^2^ A number of lifestyle factors have been associated with the risk of GC or its precursors in case-control studies among the Linqu population.^3,4,5^ Smoking has been shown to increase the risk of advanced gastric lesions and GC in a study of 564 GC cases and 1131 control cases (odds ratio [OR], 1.5; 95% CI, 1.0-2.1)^3^ and in a gastroscopic screening of 3104 residents (dysplasia [DYS] vs superficial gastritis or chronic atrophic gastritis: OR, 2.1; 95% CI, 1.4-3.0).^4^ In these studies, alcohol intake was not significantly associated with the risk of GC (OR, 0.8; 95% CI, 0.6-1.1)^3^ but was shown to be a risk factor for DYS (OR, 1.3; 95% CI, 1.0-1.8),^4^ a major precursor of GC. Among major dietary factors, increasing consumption of fresh vegetables (OR, 0.4; 95% CI, 0.3-0.6), fresh fruit (OR, 0.6; 95% CI, 0.4-0.8), garlic (OR, 0.7; 95% CI, 0.4-1.0), and vitamin C (OR, 0.5; 95% CI, 0.3-0.6) decreased the risk of GC in the previously mentioned case-control study^3^ and another analysis involving the same 564 GC cases and 1131 control participants with nutritional deficiencies in Linqu.^5^ A prospective study is needed to elucidate the associations of lifestyle factors with GC risk in high-risk populations.

In 1995, the Shandong Intervention Trial (SIT) was initiated in Linqu to evaluate the effects of *Helicobacter pylori* treatment for 2 weeks and vitamin and garlic supplementation for 7.3 years on the progression of precancerous gastric lesions and occurrence of GC.^6,7,8,9,10^ During a total follow-up of 22.3 years, in addition to *H pylori* eradication therapy, we found significantly decreased long-term risk of developing GC associated with vitamin supplementation (OR, 0.64; 95% CI, 0.46-0.91) and reduced GC mortality for those taking vitamins (hazard ratio [HR], 0.48; 95% CI, 0.31-0.75) or garlic supplements (HR, 0.66; 95% CI, 0.43-0.999), suggesting potential primary prevention of GC by lifestyle interventions.^10^ However, before nutritional supplementation can be translated to the community level, identifying the appropriate population subgroups for intervention is still warranted to optimize the potential beneficial effects. Given our ongoing efforts to promote precision prevention and control of GC, it will be important to illuminate the effects of intervention strategies on subgroups based on lifestyle factors and to identify any effect modifications by lifestyle factors on GC prevention resulting from long-term nutritional supplementation. Our interest in the potential effect modification of lifestyle factors on GC prevention strategies was further increased by reports that smoking, alcohol consumption, and diet alter gastric microbiota composition.^11,12,13,14^ Based on the SIT, we evaluated the effects of vitamin supplementation and garlic supplementation on GC incidence and mortality in subgroups based on major lifestyle factors. The association of lifestyle factors with GC incidence and mortality was examined, and the interactions between lifestyle factors and the 2 nutritional supplementations were further tested. In addition, the association of lifestyle factors with the progression of gastric lesions was assessed.

## Methods

Details of the SIT have been described previously^6,7,8^ and in our recent study.^10^ We conducted an unplanned secondary analysis of the trial, including the associations with lifestyle factors in a longitudinal study and the modifications of lifestyle factors on the effects of vitamin and garlic supplementations on GC incidence and mortality. The secondary analysis was approved by the institutional review board of Peking University Cancer Hospital. Informed consent was waived for this secondary analysis because the study posed minimal risk using existing data. This study was reported following the Consolidated Standards of Reporting Trials (CONSORT) reporting guideline.^15^

### Study Population

**Figure 1.**
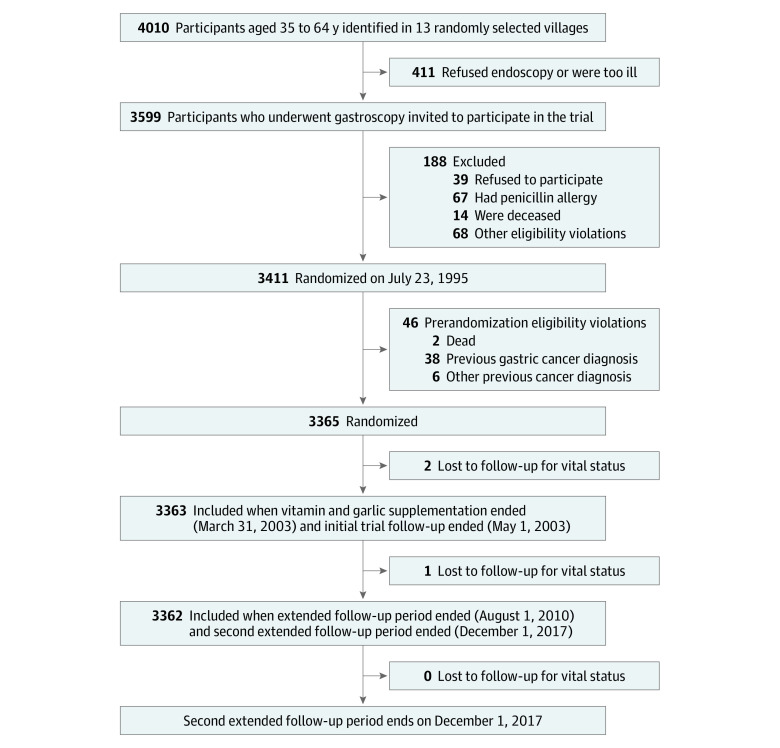
Study Flow Diagram

The study flow diagram and participants’ eligibility criteria are shown in Figure 1. A total of 3365 eligible participants were randomly assigned to 3 interventions, including vitamin and garlic supplementation, *H pylori* treatment for those with *H pylori*, or placebo.^6^ The participants were observed until 2017, with a total of 22.3 years since trial randomization. Only 3 participants (<0.01%) were lost to follow-up for vital status during the follow-up period (Figure 1).

### Outcomes

The primary outcomes were GC incidence and mortality. We ascertained GC incidence from scheduled gastroscopies, cancer registries, or autopsy reports, which were confirmed by reviewing medical records. Scheduled endoscopies were conducted in 1999 and 2003 for all participants and in 2005 and 2007 for those diagnosed with intestinal metaplasia or DYS, respectively, during their 2003 endoscopy. In addition, repeated endoscopies were conducted every 6 months to 1 year between 2008 and 2017 for those diagnosed with moderate or severe DYS at any biopsy site or with mild DYS at 2 or more sites in 2003.^10^ Information on GC death was obtained from the reporting system managed by the Chinese Center for Disease Control and Prevention, which integrates death certificates from hospitals as well as from police and judicial departments in Linqu. Active clinical follow-up has also been conducted by village physicians and staff from Peking University Cancer Hospital.^10^

In addition, we examined the progression of gastric lesions (ie, progression or nonprogression) from 1995 to 2003 as a secondary outcome. This was assessed based on the 7.3-year follow-up only because histology diagnosis of gastric lesions with scheduled endoscopy was only available through 2003 for all participants. We were not able to examine progression of gastric lesions (progression vs nonprogression) through 22.3 years’ follow-up. Each participant was assigned a severity score in 1995 and 2003, with 0 indicating normal, 1 indicating superficial gastritis, 2 indicating mild chronic atrophic gastritis, 3 indicating severe chronic atrophic gastritis, 4 indicating superficial intestinal metaplasia, 5 indicating deep intestinal metaplasia, 6 indicating mild DYS, 7 indicating moderate DYS, 8 indicating severe DYS, and 9 indicating GC. If the score in 2003 was higher than that in 1995, the participant was classified as having progression of gastric lesions.

### Assessment of Major Lifestyle Factors

In 1994, all participants who were identified in the census were interviewed for baseline characteristics by trained staff. Information on lifestyle factors, including smoking, alcohol intake, and major dietary factors, was collected for all participants using a structured questionnaire. The participants were asked about their history of tobacco use and alcohol intake. Based on their answers, the dichotomous categories of smoking status (ever vs never) and alcohol consumption (ever vs never) were classified. A total of 112 participants (3.3%) had missing information on both smoking and alcohol intake.

The questionnaire evaluated yearly consumption (in kilograms per year) of multiple food items, including grain, meat, fresh vegetables and fruits, and total vegetables and fruits. For all analyses of dietary factors, participants were dichotomized based on the median yearly intake of each item among all participants. A total of 80 participants (2.4%) had missing information on each dietary factor.

### Statistical Analysis

For the analyses of primary outcomes, we first examined the associations of smoking, alcohol intake, and major dietary factors with the cumulative incidence and mortality of GC. The association of vitamin and garlic supplementation with GC incidence and mortality were then examined in the subgroups of these lifestyle factors. Conditional logistic regression models stratified on baseline histopathology were used to estimate the ORs and corresponding 95% CIs for the risk of GC cumulative incidence, given that many GC cases were diagnosed at scheduled gastroendoscopies. Associations with GC mortality were analyzed by Cox regression models, with HRs and 95% CIs estimated. Multivariable-adjusted regression analyses were performed with adjustment for other potential confounders, including age, sex, and baseline histology.^16^ History of ever using alcohol (ever vs never) and history of ever smoking (ever vs never) were also adjusted for in the models, except when they were being examined as the main exposure factor or main effect modifier because of the association with GC or its precursors based on case-control studies in Linqu.^3,4,5^ For the analyses of the association of smoking or alcohol intake with GC, we also controlled for vitamin supplementation, garlic supplementation, and *H pylori* treatment because these interventions were associated with decreased GC incidence and/or mortality.^10^

We examined the associations of vitamin and garlic supplementation with GC incidence and mortality in subgroups by smoking, alcohol intake, and consumption of each dietary factor, dichotomized by the median yearly intake among all participants. *P* values for interactions were calculated by adding the interaction term between each examined lifestyle factor and the intervention in the regression models in addition to the indicators of the 2 items being analyzed.

For the secondary outcomes, we examined the association between lifestyle factors and the 2 interventions on the progression of gastric lesions (progression vs nonprogression) using conditional logistic regression models. The previously described analyses of associations and interaction analyses were conducted.

Post hoc analyses of statistical power were calculated using R software version 3.5.2 (R Project for Statistical Computing). All other analyses were performed using the intention-to-treat approach and were conducted using SAS software version 9.4 (SAS Institute). A 2-tailed *P* < .05 was considered statistically significant. Statistical analysis was conducted from March to May 2019.

## Results

A total of 3365 participants were included in the study, with a mean (SD) age of 47.1 (9.2) years and 1639 (48.7%) women. Distribution of study participants and major lifestyle factors is shown in Table 1. During the follow-up, we identified 151 (4.5%; 95% CI, 3.8%-5.3%) GC cases and 94 (2.8%; 95% CI, 2.3%-3.4%) GC deaths.^10^ After those with missing data on major independent variables or covariates were excluded from multivariable models, 3237 participants (96.2%) remained for the analyses of smoking and alcohol drinking, and 3168 (94.1%) remained for the analyses of dietary factors. We did not find significant differences in the distribution of major characteristics between the remaining and overall trial participants (eTable 1 in Supplement 1). The number of GC cases and deaths per subgroup after excluding participants who were missing information for each subgroup variable is shown in Table 2.

**Table 1.** Distribution in Lifestyle Factors at Baseline

Factor	Supplementation group, %			
	Vitamin		Garlic	
	Active (n = 1677)	Placebo (n = 1688)	Active (n = 1678)	Placebo (n = 1687)
Smoking				
Never	914 (54.5)	920 (54.5)	909 (54.1)	925 (54.8)
Ever	703 (41.9)	716 (42.4)	706 (42.1)	713 (42.3)
Missing	60 (3.6)	52 (3.1)	63 (3.8)	49 (2.9)
Alcohol drinking				
Never	864 (51.5)	891 (52.8)	875 (52.1)	880 (52.2)
Ever	753 (44.9)	745 (44.1)	740 (44.1)	758 (44.9)
Missing	60 (3.6)	52 (3.1)	63 (3.8)	49 (2.9)
Dietary factors				
Grain, kg/y^a^				
<225	764 (45.6)	732 (43.4)	732 (43.6)	764 (45.3)
≥225	877 (52.3)	912 (54.0)	912 (54.4)	877 (52.0)
Missing	36 (2.1)	44 (2.6)	34 (2.0)	46 (2.7)
Meat, kg/y^a^				
<8	825 (49.2)	804 (47.6)	807 (48.1)	822 (48.7)
≥8	816 (48.7)	840 (49.8)	837 (49.9)	819 (48.5)
Missing	36 (2.1)	44 (2.6)	34 (2.0)	46 (2.8)
Total vegetables and fruit, kg/y^a^				
<92	826 (49.3)	816 (48.3)	812 (48.4)	830 (49.2)
≥92	815 (48.6)	828 (49.1)	832 (49.6)	811 (48.1)
Missing	36 (2.1)	44 (2.6)	34 (2.0)	46 (2.7)
Total fresh vegetables and fruit, kg/y^a^				
<81	819 (48.8)	821 (48.6)	809 (48.2)	831 (49.3)
≥81	822 (49.0)	823 (48.8)	835 (49.8)	810 (48.0)
Missing	36 (2.2)	44 (2.6)	34 (2.0)	46 (2.7)

^a^ Levels correspond to median distribution of intake between the 2 groups.

**Table 2.** Associations of 2 Nutritional Supplementations With Gastric Cancer Incidence and Mortality, Stratified by Lifestyle Factors

Factor	Gastric cancer incidence				Gastric cancer mortality			
	No./total No. (%)^a^		OR (95% CI)^b^	*P* value for interaction^c^	No./total No. (%)^a^		HR (95% CI)^d^	*P* value for interaction^c^
	Placebo	Active			Placebo	Active		
**Vitamin supplementation**								
Overall	89/1627 (5.5)	58/1610 (3.6)	0.64 (0.46-0.91)	NA	61/1627 (3.7)	29/1610 (1.8)	0.48 (0.31-0.75)	NA
Smoking								
Ever	58/713 (8.1)	39/698 (5.6)	0.66 (0.43-1.01)	.82	43/713 (6.0)	22/698 (3.2)	0.52 (0.31-0.87)	.50
Never	31/914 (3.4)	19/912 (2.1)	0.60 (0.34-1.08)		18/914 (2.0)	7/912 (0.8)	0.35 (0.15-0.85)	
Alcohol								
Ever	55/741 (7.4)	35/747 (4.7)	0.61 (0.39-0.96)	.72	41/741 (5.5)	18/747 (2.4)	0.44 (0.25-0.76)	.54
Never	34/886 (3.8)	23/863 (2.7)	0.70 (0.40-1.20)		20/886 (2.3)	11/863 (1.3)	0.58 (0.28-1.21)	
Grain, kg/y^e^								
<225	36/700 (5.1)	26/732 (3.6)	0.68 (0.40-1.15)	.69	27/700 (3.9)	9/732 (1.2)	0.31 (0.15-0.67)	.24
≥225	51/890 (5.7)	30/846 (3.5)	0.58 (0.36-0.94)		34/890 (3.8)	18/846 (2.1)	0.56 (0.32-0.99)	
Meat, kg/y^e^								
<8	43/776 (5.5)	29/801 (3.6)	0.61 (0.37-1.01)	.84	30/776 (3.9)	12/801 (1.5)	0.39 (0.20-0.76)	.49
≥8	44/814 (5.4)	27/777 (3.5)	0.67 (0.40-1.10)		31/814 (3.8)	15/777 (1.9)	0.53 (0.28-0.98)	
Total vegetables and fruits, kg/y^e^								
<92	47/785 (6.0)	28/796 (3.5)	0.58 (0.36-0.96)	.70	31/785 (3.9)	12/796 (1.5)	0.37 (0.19-0.73)	.50
≥92	40/805 (5.0)	28/782 (3.7)	0.68 (0.41-1.14)		30/805 (3.7)	15/782 (1.9)	0.51 (0.27-0.95)	
Total fresh vegetables and fruits, kg/y^e^								
<81	47/789 (6.0)	26/788 (3.3)	0.55 (0.34-0.92)	.49	31/789 (3.9)	13/788 (1.6)	0.41 (0.22-0.79)	.79
≥81	40/801 (5.0)	30/790 (3.8)	0.72 (0.44-1.18)		30/801 (3.7)	14/790 (1.7)	0.47 (0.25-0.89)	
**Garlic supplementation**								
Overall	81/1631 (5.0)	66/1606 (4.1)	0.81 (0.57-1.13)	NA	54/1631 (3.3)	36/1606 (2.2)	0.66 (0.43-0.999)	NA
Smoking								
Ever	54/711 (7.6)	43/700 (6.1)	0.78 (0.51-1.20)	.84	38/711 (5.3)	27/700 (3.9)	0.70 (0.43-1.15)	.58
Never	27/920 (2.9)	23/906 (2.5)	0.86 (0.49-1.52)		16/920 (1.7)	9/906 (1.0)	0.51 (0.23-1.17)	
Alcohol								
Ever	46/756 (6.1)	44/732 (6.0)	0.99 (0.64-1.54)	.16	31/756 (4.1)	28/732 (3.8)	0.92 (0.55-1.54)	.03
Never	35/875 (4.0)	22/874 (2.5)	0.62 (0.36-1.07)		23/875 (2.6)	8/874 (0.9)	0.33 (0.15-0.75)	
Grain, kg/y^e^								
<225	31/731 (4.2)	31/701 (4.4)	1.05 (0.62-1.76)	.31	21/731 (2.9)	15/701 (2.1)	0.71 (0.36-1.39)	.82
≥225	47/859 (5.5)	34/877 (3.9)	0.72 (0.45-1.15)		32/859 (3.7)	20/877 (2.3)	0.64 (0.37-1.12)	
Meat, kg/y^e^								
<8	39/797 (4.9)	33/780 (4.2)	0.85 (0.52-1.38)	.92	25/797 (3.1)	17/780 (2.2)	0.68 (0.36-1.26)	.84
≥8	39/793 (4.9)	32/798 (4.0)	0.81 (0.49-1.32)		28/793 (3.5)	18/798 (2.3)	0.62 (0.34-1.13)	
Total vegetables and fruits, kg/y^e^								
<92	43/796 (5.4)	32/785 (4.1)	0.76 (0.47-1.22)	.63	28/796 (3.5)	15/785 (1.9)	0.53 (0.28-1.00)	.41
≥92	35/794 (4.4)	33/793 (4.2)	0.88 (0.53-1.45)		25/794 (3.1)	20/793 (2.5)	0.78 (0.43-1.40)	
Total fresh vegetables and fruits, kg/y^e^								
<81	42/795 (5.3)	31/782 (4)	0.75 (0.46-1.22)	.64	29/795 (3.6)	15/782 (1.9)	0.52 (0.28-0.96)	.34
≥81	36/795 (4.5)	34/796 (4.3)	0.88 (0.54-1.43)		24/795 (3)	20/796 (2.5)	0.80 (0.44-1.45)	

Abbreviations: HR, hazard ratio; NA, not applicable; OR, odds ratio.

^a^ Number of participants with an event divided by total number of participants. Those with missing data on the examined independent factor or other covariates were excluded from the multivariable models.

^b^ Logistic regression adjusted for baseline histology, age, sex, history of ever using alcohol, and history of ever smoking.

^c^ *P* values for interactions were calculated by adding the interaction term between 2 items in the regression models in addition to the indicators of the 2 items being analyzed.

^d^ Cox regression adjusted for baseline histology, age, sex, history of ever using alcohol, and history of ever smoking.

^e^ Levels correspond to median distribution of intake between 2 groups.

### Associations Between Lifestyle Factors and GC Risk

**Figure 2.**
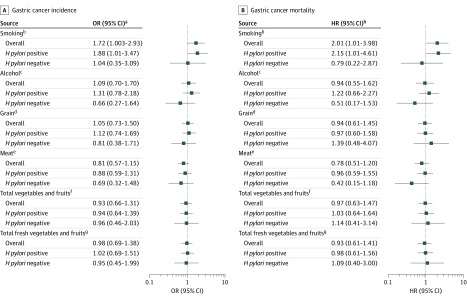
Associations Between Lifestyle Factors and Gastric Cancer Incidence and Mortality HR indicates hazard ratio; OR, odds ratio. ^a^Logistic regression adjusted for baseline histology, age, sex, history of ever using alcohol, history of ever smoking, vitamin supplementation (treatment or placebo), garlic supplementation (treatment or placebo), and *H pylori* treatment (*H pylori* treatment, placebo, or no *H pylori*). ^b^Ever smoking vs never smoking. ^c^Ever using alcohol vs never using alcohol. ^d^Consuming at least 225 kg/y vs less than 225 kg/y (levels correspond to median distribution of intake between the 2 groups). ^e^Consuming at least 8 kg/y vs less than 8 kg/y (levels correspond to median distribution of intake between the 2 groups). ^f^Consuming at least 92 kg/y vs less than 92 kg/y (levels correspond to median distribution of intake between the 2 groups). ^g^Consuming at least 81 kg/y vs less than 81 kg/y (levels correspond to median distribution of intake between the 2 groups). ^h^Cox regression adjusted for baseline histology, age, sex, history of ever using alcohol, history of ever smoking, vitamin supplementation (treatment or placebo), garlic supplementation (treatment or placebo), and *H pylori* treatment (*H pylori* treatment, placebo, or no *H pylori*).

Our analyses showed that smoking was associated with an increased risk of GC incidence (multivariable-adjusted OR, 1.72; 95% CI, 1.003-2.93) and GC mortality (HR, 2.01; 95% CI, 1.01-3.98) (Figure 2). The associations were significant only among participants with *H pylori*, for whom the OR for GC incidence was 1.88 (95% CI, 1.01-3.47) and the HR for GC mortality was 2.15 (95% CI, 1.01-4.61). In contrast, the associations were not significant among participants without *H pylori* (Figure 2). We did not find any significant associations between alcohol consumption and risk of GC incidence and mortality overall or in specific *H pylori* infection groups. Similarly, major dietary factors were not significantly associated with risk of GC incidence and mortality (Figure 2).

### Interaction Between Lifestyle Factors and Nutritional Supplementation on GC Incidence and Mortality

In the subgroup analyses, we did not find any significant effect modifications by smoking or dietary factors on the associations of nutritional supplementation with risk of GC incidence and mortality (Table 2). However, garlic supplementation was associated with reduced mortality among those who never drank alcohol (HR, 0.33; 95% CI, 0.15-0.75) but not among those who ever drank alcohol (HR, 0.92; 95% CI, 0.55-1.54; *P* for interaction = .03). No significant interactions between vitamin supplementation and alcohol intake were found on risk of GC incidence or mortality (Table 2).

We further examined interactions stratified by baseline *H pylori* infection status. The significant interactions between alcohol intake and garlic supplementation on GC mortality were only seen among participants with *H pylori*, among whom the protective effect was significant for those who never drank alcohol (HR, 0.31; 95% CI, 0.12-0.78) but not for those who ever drank alcohol (HR, 0.91; 95% CI, 0.52-1.60; *P *for interaction = .04) (Table 3).

**Table 3.** Association of Garlic Supplementation With Gastric Cancer Incidence and Mortality Stratified by Alcohol Intake Among Participants With and Without *Helicobacter pylori*

Factor	Gastric cancer incidence				Gastric cancer mortality			
	No./total No.^a^		OR (95% CI)^b^	*P* value for interaction^c^	No./total No.^a^		HR (95% CI)^d^	*P* value for interaction^c^
	Placebo	Active			Placebo	Active		
**With *H pylori***								
Overall	63/1092 (5.8)	53/1080 (4.9)	0.84 (0.57-1.23)	NA	44/1092 (4.0)	30/1080 (2.8)	0.66 (0.42-1.06)	NA
Alcohol								
Ever	38/501 (7.6)	36/489 (7.4)	0.99 (0.61-1.62)	.27	26/501 (5.2)	24/489 (4.9)	0.91 (0.52-1.60)	.04
Never	25/591 (4.2)	17/591 (2.9)	0.64 (0.34-1.22)		18/591 (3.0)	6/591 (1.o)	0.31 (0.12-0.78)	
**No *H pylori***								
Overall	18/539 (3.3)	13/526 (2.5)	0.73 (0.35-1.53)	NA	10/539 (1.9)	6/526 (1.1)	0.60 (0.21-1.67)	NA
Alcohol								
Ever	8/255 (3.1)	8/243 (3.3)	0.94 (0.33-2.70)	.31	5/255 (2.0)	4/243 (1.6)	0.73 (0.19-2.82)	.45
Never	10/284 (3.5)	5/283 (1.8)	0.45 (0.15-1.38)		5/284 (1.8)	2/283 (0.7)	0.41 (0.07-2.38)	

Abbreviations: HR, hazard ratio; NA, not applicable; OR, odds ratio.

^a^ Number of participants with an event divided by total number of participants. Those with missing data on the examined independent factor or other covariates were excluded from the multivariable models.

^b^ Logistic regression adjusted for baseline histology, age, sex, history of ever using alcohol, and history of ever smoking.

^c^ *P* values for interactions were calculated by adding the interaction term between 2 items in the regression models in addition to the indicators of the 2 items being analyzed.

^d^ Cox regression adjusted for baseline histology, age, sex, history of ever using alcohol, and history of ever smoking.

### Interaction Between Lifestyle Factors and Nutritional Supplementations on Gastric Lesion Progression

Based on gastroendoscopy and histology diagnosis in 1995 and 2003, 1261 of 2942 participants (42.9%) had progression of gastric lesions. We found statistically significant interactions between vitamin supplementation and total fresh vegetable and fruit intake (<81 kg/y: OR, 0.80; 95% CI, 0.64-1.01; ≥81 kg/y: OR, 1.17; 95% CI, 0.94-1.46; *P *for interaction = .02) on the progression of gastric lesions (eTable 2 in Supplement 1).

Further interaction analyses by baseline *H pylori* infection status found significant interactions between vitamin supplementation and total vegetable and fruit intake (<92 kg/y: OR, 0.56; 95% CI, 0.36-0.86; ≥92 kg/y: OR, 1.42; 95% CI, 0.95-2.13; *P* for interaction = .003) and total fresh vegetable and fruit intake (<81 kg/y: OR, 0.53; 95% CI, 0.35-0.82; ≥81 kg/y: OR, 1.48; 95% CI, 0.98-2.22; *P *for interaction = .001) only among participants without *H pylori* infection (eTable 3 in Supplement 1).

## Discussion

Based on a total follow-up of 22.3 years in the SIT, smoking was associated with an increased risk of GC incidence and mortality, particularly among participants with *H pylori* infection. Alcohol intake was not associated with risk of GC incidence or mortality but may be a modifier for the effect of garlic supplementation on GC prevention, with greater beneficial effects on GC, particularly among those who do not drink alcohol.

Among the major lifestyle factors, smoking is an established risk factor for GC in large prospective studies of western populations.^17,18,19^ The association between alcohol intake and GC risk has been unclear. Although an association between heavy alcohol drinking and increased GC risk was reported by Tramacere et al^20^ and Rota et al,^21^ another meta-analysis^22^and large prospective cohort study^23^ did not report this association. Neither smoking nor alcohol intake was reported as a major contributing factor for GC in 2 previous studies based on high-risk populations in China, including a prospective study^24^ and a case-control study.^25^ Previous case-control studies based on the Linqu population supported our finding that smoking, but not alcohol intake, was a risk factor for GC.^3^ Consistent with findings from case-control studies, we conducted a large prospective analysis with follow-up of 2 decades, which again supported smoking as a risk factor for GC incidence and mortality. The increased exposure to potent carcinogens and N-nitroso compounds when smoking may partly help to explain the increased risk of GC among those who smoke.^26^ In a Korean case-cohort study,^27^ heavy alcohol intake (≥7 times a week) was associated with an increased risk of GC among those not infected with *H pylori*. However, we did not find such an association for alcohol intake. In previous case-control studies of the Linqu population,^3^ high consumption of total fruits and vegetables was associated with a decreased risk of GC. Previous large cohort studies and case-control studies from other populations reported heterogeneous associations between GC and consumption of fruit and vegetables or fresh fruit.^28,29,30,31,32^ In this prospective analysis, we did not find any significant association of total fruit and vegetable intake and other dietary factors with risk of GC incidence or mortality.

Allyl sulfur compounds from garlic are effective against carcinogenesis by inhibiting the activation of carcinogens, modulating carcinogen metabolism, and inhibiting formation of DNA adducts with carcinogens, among other mechanisms.^33^ Alcohol produces harmful effects in the stomach, causing direct damage to DNA^14^ and inflammation.^34^ Although our study failed to find significant associations between alcohol intake and GC risk, alcohol intake may modify the effect of garlic supplementation, with the protective effect of garlic supplementation on GC mortality seen particularly among those who did not drink alcohol. Similarly, a 2019 case-control study^35^ reported an interaction of garlic consumption with alcohol drinking on esophageal cancer in a population in China. Garlic supplementation may take effect by protecting gastric mucosa against damage and inflammatory response induced by alcohol, and the hypothesis was confirmed by animal studies.^34,36,37^ Furthermore, alteration of gastric microbiota has been observed in GC and gastric lesions, indicating microbial dysbiosis in gastric carcinogenesis.^38,39^ Considering that dysbiosis of microbiota could be induced by alcohol as well as garlic supplementation, it remains to be elucidated whether the exploration of microbial dysbiosis can help to explain the interaction between alcohol intake and garlic supplementation on GC risk.^12,14,40^

A greater preventive effect of vitamin supplementation was seen among those with low fresh vegetable and fruit intake. The micronutrients from fruits and vegetables, such as selenium, beta carotene, and vitamin C, may contribute to the protective effect for gastric mucosa by anti-inflammatory damage.^41^ Vitamin C was also associated with decreased risk of GC in studies of high-risk^8,9,42^ and general populations.^43,44^ Notably, in our population, the significant interaction was only seen for gastric lesion progression but not for GC incidence or mortality. The reason for this is unclear. However, during gastric carcinogenesis, the pH level in the stomach decreases initially but increases with gastric lesion progression when vitamin C, ie, ascorbic acid, is converted to the less-active form of dehydroascorbic acid^45^ with weaker protection.

In addition to vitamin and garlic supplementation, 1-time *H pylori* treatment for 2 weeks was an intervention in the SIT. During gastric carcinogenesis, *H pylori* infection is recognized as playing a crucial role in the initial steps of carcinogenesis by causing enhanced inflammation and histological changes.^46^ Previous studies did not show evidence on the differential associations of *H pylori* infection with GC according to smoking, alcohol intake, and dietary factors.^21^ Indeed, we have reported that smoking and alcohol intake are associated with *H pylori* eradication failure.^47^ Therefore, we did not seek to examine interactions between *H pylori* treatment and major lifestyle factors in our study.

### Limitations

We acknowledge several limitations. First, we had a modest sample size, and statistical power for some analyses may be limited, as shown in eTable 4 in Supplement 1. We do not have the information on cardia or noncardia cancer for all GC cases, which precluded an analysis of cardia GC separately. Second, detailed information on lifestyle factors, such as the frequency and volume of alcohol intake, duration and pack-year of cigarette smoking, and information on specific diet, was not available, which prevented us from examining lifestyle factors in closer detail. Third, we have no relevant data regarding lifestyle changes during the follow-up period and were not able to examine updated lifestyle factors. However, it is unlikely that such alterations would occur differentially in the intervention and control groups. Furthermore, our study was based on a high-risk rural population with nutritional deficiencies, and the extrapolation to general populations requires cautious interpretation.

## Conclusions

In this study, based on a well-defined high-risk population with long-term follow-up, smoking was associated with increased risk of GC incidence and mortality. Alcohol intake may be an effect modifier for the protective effect of garlic supplementation on GC. Our findings provide new insights into lifestyle interventions for GC prevention, suggesting that mass GC prevention strategies may need to be tailored to population subgroups to maximize the potential beneficial effects.

## Notes

Supplement 1. **eTable 1. **Assessment of Missing Data **eTable 2. **Effects of Vitamin Supplementation on Gastric Lesion Progression Stratified by Lifestyle Factors **eTable 3. **Effects of Vitamin Supplementation on the Progression of Gastric Lesions, Stratified by Consumption of Vegetables and Fruit Among Participants With and Without *H pylori* Infection **eTable 4. **Statistical Power for Association and Subgroup Analyses

Supplement 2. **Data Sharing Statement**

## References

[1] Bray F, Ferlay J, Soerjomataram I, Siegel RL, Torre LA, Jemal A Global cancer statistics 2018: GLOBOCAN estimates of incidence and mortality worldwide for 36 cancers in 185 countries. CA Cancer J Clin. 2018;68(6):394-424. doi:10.3322/caac.21492 30207593

[2] You WC, Blot WJ, Li JY, Precancerous gastric lesions in a population at high risk of stomach cancer. Cancer Res. 1993;53(6):1317-1321. 8443811

[3] You WC, Blot WJ, Chang YS, Diet and high risk of stomach cancer in Shandong, China. Cancer Res. 1988;48(12):3518-3523. 3370645

[4] Kneller RW, You WC, Chang YS, Cigarette smoking and other risk factors for progression of precancerous stomach lesions. J Natl Cancer Inst. 1992;84(16):1261-1266. doi:10.1093/jnci/84.16.1261 1640486

[5] You WC, Blot WJ, Chang YS, Allium vegetables and reduced risk of stomach cancer. J Natl Cancer Inst. 1989;81(2):162-164. doi:10.1093/jnci/81.2.162 2909758

[6] Gail MH, You WC, Chang YS, Factorial trial of three interventions to reduce the progression of precancerous gastric lesions in Shandong, China: design issues and initial data. Control Clin Trials. 1998;19(4):352-369. doi:10.1016/S0197-2456(98)00016-6 9683311

[7] You WC, Brown LM, Zhang L, Randomized double-blind factorial trial of three treatments to reduce the prevalence of precancerous gastric lesions. J Natl Cancer Inst. 2006;98(14):974-983. doi:10.1093/jnci/djj264 16849680

[8] Ma JL, Zhang L, Brown LM, Fifteen-year effects of *Helicobacter pylori*, garlic, and vitamin treatments on gastric cancer incidence and mortality. J Natl Cancer Inst. 2012;104(6):488-492. doi:10.1093/jnci/djs003 22271764 PMC3309129

[9] Li WQ, Ma JL, Zhang L, Effects of *Helicobacter pylori* treatment on gastric cancer incidence and mortality in subgroups. J Natl Cancer Inst. 2014;106(7):dju116. doi:10.1093/jnci/dju116 24925350 PMC4067110

[10] Li W-Q, Zhang J-Y, Ma J-L, Effects of *Helicobacter pylori* treatment and vitamin and garlic supplementation on gastric cancer incidence and mortality: follow-up of a randomized intervention trial. BMJ. 2019;366:l5016. doi:10.1136/bmj.l5016 31511230 PMC6737461

[11] Vogtmann E, Flores R, Yu G, Association between tobacco use and the upper gastrointestinal microbiome among Chinese men. Cancer Causes Control. 2015;26(4):581-588. doi:10.1007/s10552-015-0535-2 25701246 PMC4852095

[12] Qamar N, Castano D, Patt C, Chu T, Cottrell J, Chang SL Meta-analysis of alcohol induced gut dysbiosis and the resulting behavioral impact. Behav Brain Res. 2019;376:112196. doi:10.1016/j.bbr.2019.112196 31476330

[13] Valdes AM, Walter J, Segal E, Spector TD Role of the gut microbiota in nutrition and health. BMJ. 2018;361:k2179. doi:10.1136/bmj.k2179 29899036 PMC6000740

[14] Capurso G, Lahner E The interaction between smoking, alcohol and the gut microbiome. Best Pract Res Clin Gastroenterol. 2017;31(5):579-588. doi:10.1016/j.bpg.2017.10.006 29195678

[15] Moher D, Schulz KF, Altman DG The CONSORT statement: revised recommendations for improving the quality of reports of parallel-group randomised trials. Lancet. 2001;357(9263):1191-1194. doi:10.1016/S0140-6736(00)04337-3 11323066

[16] You WC, Li JY, Blot WJ, Evolution of precancerous lesions in a rural Chinese population at high risk of gastric cancer. Int J Cancer. 1999;83(5):615-619. doi:10.1002/(SICI)1097-0215(19991126)83:5<615::AID-IJC8>3.0.CO;2-L 10521796

[17] Steevens J, Schouten LJ, Goldbohm RA, van den Brandt PA Alcohol consumption, cigarette smoking, and risk of subtypes of oesophageal and gastric cancer: a prospective cohort study. Gut. 2010;59(1):39-48. doi:10.1136/gut.2009.191080 19828467

[18] Sjödahl K, Lu Y, Nilsen TI, Smoking and alcohol drinking in relation to risk of gastric cancer: a population-based, prospective cohort study. Int J Cancer. 2007;120(1):128-132. doi:10.1002/ijc.22157 17036324

[19] Freedman ND, Abnet CC, Leitzmann MF, A prospective study of tobacco, alcohol, and the risk of esophageal and gastric cancer subtypes. Am J Epidemiol. 2007;165(12):1424-1433. doi:10.1093/aje/kwm051 17420181

[20] Tramacere I, Negri E, Pelucchi C, A meta-analysis on alcohol drinking and gastric cancer risk. Ann Oncol. 2012;23(1):28-36. doi:10.1093/annonc/mdr135 21536659

[21] Rota M, Pelucchi C, Bertuccio P, Alcohol consumption and gastric cancer risk: a pooled analysis within the StoP project consortium. Int J Cancer. 2017;141(10):1950-1962. doi:10.1002/ijc.30891 28718913

[22] Tramacere I, Pelucchi C, Bagnardi V, A meta-analysis on alcohol drinking and esophageal and gastric cardia adenocarcinoma risk. Ann Oncol. 2012;23(2):287-297. doi:10.1093/annonc/mdr136 21551004

[23] Wang S, Freedman ND, Loftfield E, Hua X, Abnet CC Alcohol consumption and risk of gastric cardia adenocarcinoma and gastric noncardia adenocarcinoma: a 16-year prospective analysis from the NIH-AARP diet and health cohort. Int J Cancer. 2018;143(11):2749-2757. doi:10.1002/ijc.31740 29992560 PMC7579679

[24] Tran GD, Sun XD, Abnet CC, Prospective study of risk factors for esophageal and gastric cancers in the Linxian general population trial cohort in China. Int J Cancer. 2005;113(3):456-463. doi:10.1002/ijc.20616 15455378

[25] Gao Y, Hu N, Han XY, Risk factors for esophageal and gastric cancers in Shanxi Province, China: a case-control study. Cancer Epidemiol. 2011;35(6):e91-e99. doi:10.1016/j.canep.2011.06.006 21846596 PMC3215853

[26] Jakszyn P, Bingham S, Pera G, Endogenous versus exogenous exposure to N-nitroso compounds and gastric cancer risk in the European Prospective Investigation into Cancer and Nutrition (EPIC-EURGAST) study. Carcinogenesis. 2006;27(7):1497-1501. doi:10.1093/carcin/bgl019 16571648

[27] Ma SH, Jung W, Weiderpass E, Impact of alcohol drinking on gastric cancer development according to *Helicobacter pylori* infection status. Br J Cancer. 2015;113(9):1381-1388. doi:10.1038/bjc.2015.333 26379079 PMC4815794

[28] Shimazu T, Wakai K, Tamakoshi A, ; Research Group for the Development and Evaluation of Cancer Prevention Strategies in Japan Association of vegetable and fruit intake with gastric cancer risk among Japanese: a pooled analysis of four cohort studies. Ann Oncol. 2014;25(6):1228-1233. doi:10.1093/annonc/mdu115 24618149

[29] Jeurnink SM, Büchner FL, Bueno-de-Mesquita HB, Variety in vegetable and fruit consumption and the risk of gastric and esophageal cancer in the European Prospective Investigation into Cancer and Nutrition. Int J Cancer. 2012;131(6):E963-E973. doi:10.1002/ijc.27517 22392502

[30] Gonzalez CA, Lujan-Barroso L, Bueno-de-Mesquita HB, Fruit and vegetable intake and the risk of gastric adenocarcinoma: a reanalysis of the European Prospective Investigation into Cancer and Nutrition (EPIC-EURGAST) study after a longer follow-up. Int J Cancer. 2012;131(12):2910-2919. doi:10.1002/ijc.27565 22473701

[31] Steevens J, Schouten LJ, Goldbohm RA, van den Brandt PA Vegetables and fruits consumption and risk of esophageal and gastric cancer subtypes in the Netherlands Cohort Study. Int J Cancer. 2011;129(11):2681-2693. doi:10.1002/ijc.25928 21960262

[32] González CA, Pera G, Agudo A, Fruit and vegetable intake and the risk of stomach and oesophagus adenocarcinoma in the European Prospective Investigation into Cancer and Nutrition (EPIC-EURGAST). Int J Cancer. 2006;118(10):2559-2566. doi:10.1002/ijc.21678 16380980

[33] Milner JA A historical perspective on garlic and cancer. J Nutr. 2001;131(3s):1027S-1031S. doi:10.1093/jn/131.3.1027S 11238810

[34] Lee IC, Baek HS, Kim SH, Effect of diallyl disulfide on acute gastric mucosal damage induced by alcohol in rats. Hum Exp Toxicol. 2015;34(3):227-239. doi:10.1177/0960327114537095 24972622

[35] Jin ZY, Wallar G, Zhou JY, Consumption of garlic and its interactions with tobacco smoking and alcohol drinking on esophageal cancer in a Chinese population. Eur J Cancer Prev. 2019;28(4):278-286. doi:10.1097/CEJ.0000000000000456 30001285 PMC6329680

[36] Zeng T, Zhang CL, Song FY, The activation of HO-1/Nrf-2 contributes to the protective effects of diallyl disulfide (DADS) against ethanol-induced oxidative stress. Biochim Biophys Acta. 2013;1830(10):4848-4859. doi:10.1016/j.bbagen.2013.06.028 23816986

[37] Khosla P, Karan RS, Bhargava VK Effect of garlic oil on ethanol induced gastric ulcers in rats. Phytother Res. 2004;18(1):87-91. doi:10.1002/ptr.1349 14750208

[38] Ferreira RM, Pereira-Marques J, Pinto-Ribeiro I, Gastric microbial community profiling reveals a dysbiotic cancer-associated microbiota. Gut. 2018;67(2):226-236. doi:10.1136/gutjnl-2017-314205 29102920 PMC5868293

[39] Coker OO, Dai Z, Nie Y, Mucosal microbiome dysbiosis in gastric carcinogenesis. Gut. 2018;67(6):1024-1032. doi:10.1136/gutjnl-2017-314281 28765474 PMC5969346

[40] Chen K, Xie K, Liu Z, Preventive effects and mechanisms of garlic on dyslipidemia and gut microbiome dysbiosis. Nutrients. 2019;11(6):E1225. doi:10.3390/nu11061225 31146458 PMC6627858

[41] Sezikli M, Çetinkaya ZA, Güzelbulut F, Effects of alpha tocopherol and ascorbic acid on *Helicobacter pylori* colonization and the severity of gastric inflammation. Helicobacter. 2012;17(2):127-132. doi:10.1111/j.1523-5378.2011.00925.x 22404443

[42] You WC, Zhang L, Gail MH, Gastric dysplasia and gastric cancer: *Helicobacter pylori*, serum vitamin C, and other risk factors. J Natl Cancer Inst. 2000;92(19):1607-1612. doi:10.1093/jnci/92.19.1607 11018097

[43] Lam TK, Freedman ND, Fan JH, Prediagnostic plasma vitamin C and risk of gastric adenocarcinoma and esophageal squamous cell carcinoma in a Chinese population. Am J Clin Nutr. 2013;98(5):1289-1297. doi:10.3945/ajcn.113.061267 24025629 PMC3798080

[44] Gonzalez CA, Riboli E Diet and cancer prevention: contributions from the European Prospective Investigation into Cancer and Nutrition (EPIC) study. Eur J Cancer. 2010;46(14):2555-2562. doi:10.1016/j.ejca.2010.07.025 20843485

[45] Woodward M, Tunstall-Pedoe H, McColl K *Helicobacter pylori* infection reduces systemic availability of dietary vitamin C. Eur J Gastroenterol Hepatol. 2001;13(3):233-237. doi:10.1097/00042737-200103000-00003 11293441

[46] Correa P Human gastric carcinogenesis: a multistep and multifactorial process: First American Cancer Society Award Lecture on Cancer Epidemiology and Prevention. Cancer Res. 1992;52(24):6735-6740. 1458460

[47] Pan KF, Zhang L, Gerhard M, A large randomised controlled intervention trial to prevent gastric cancer by eradication of *Helicobacter pylori *in Linqu County, China: baseline results and factors affecting the eradication. Gut. 2016;65(1):9-18. doi:10.1136/gutjnl-2015-309197 25986943

