# Supplementary material for: Association Between Lifestyle Factors, Vitamin and Garlic Supplementation, and Gastric Cancer Outcomes: A Secondary Analysis of a Randomized Clinical Trial
Source: JAMA Netw Open. 2020 Jun 26;3(6):e206628. doi: 10.1001/jamanetworkopen.2020.6628 (PMC7320300; doi:10.1001/jamanetworkopen.2020.6628)
Supplement: Supplement 1. — eTable 1. Assessment of Missing Data eTable 2. Effects of Vitamin Supplementation on Gastric Lesion Progression Stratified by Lifestyle Factors eTable 3. Effects of Vitamin Supplementation on the Progression of Gastric Lesions, Stratified by Consumption of Vegetables and Fruit Among Participants With and Without H pylori Infection eTable 4. Statistical Power for Association and Subgroup Analyses [file jamanetwopen-3-e206628-s001.pdf]

Supplementary Online Content

Guo Y, Li Z-X, Zhang J-Y, et al. Association between lifestyle factors, vitamin and garlic supplementation, and gastric cancer outcomes: a secondary analysis of a randomized clinical trial. *JAMA Netw Open*. 2020;3(6):e206628.
doi:10.1001/jamanetworkopen.2020.6628

eTable 1. Assessment of Missing Data
eTable 2. Effects of Vitamin Supplementation on Gastric Lesion Progression Stratified by Lifestyle Factors
eTable 3. Effects of Vitamin Supplementation on the Progression of Gastric Lesions, Stratified by Consumption of Vegetables and Fruit Among Participants With and Without *H pylori* Infection
eTable 4. Statistical Power for Association and Subgroup Analyses

This supplementary material has been provided by the authors to give readers additional information about their work.

© 2020 Guo Y et al. *JAMA Network Open*.

eTable 1. Assessment of Missing Data[a]

	Analyses of smoking and alcohol drinking				Analyses of dietary factors		
	Subjects remaining in the analyses[b] n=3237	Overall trial participants n=3365	*P*		Subjects remaining in the analyses[b] n=3168	Overall trial participants n=3365	*P*
Age	46.9 ± 9.1	47.1 ± 9.2	.46		46.9 ± 9.1	47.1 ± 9.2	.34
Sex			.61				.65
Male	1640 (50.7%)	1726 (51.3%)			1607 (50.7%)	1726 (51.3%)	
Female	1597 (49.3%)	1639 (48.7%)			1561 (49.3%)	1639 (48.7%)	
Gastric lesions			.82				.68
Normal/SG/CAG	1556 (48.1%)	1598 (47.8%)			1530 (48.3%)	1598 (47.8%)	
IM/DYS	1681 (51.9%)	1746 (52.2%)			1638 (51.7%)	1746 (52.2%)	
Vitamin supplementation			.94				.98
Active	1610 (49.7%)	1677 (49.8%)			1578 (49.8%)	1677 (49.8%)	
Placebo	1627 (50.3%)	1688 (50.2%)			1590 (50.2%)	1688 (50.2%)	
Garlic supplementation			.84				.96
Active	1606 (49.6%)	1678 (49.9%)			1578 (49.8%)	1678 (49.9%)	
Placebo	1631 (50.4%)	1687 (50.1%)			1590 (50.2%)	1687 (50.1%)	
GC incidence			.92				.96
Yes	147 (4.5%)	151 (4.5%)			143 (4.5%)	151 (4.5%)	
No	3090 (95.5%)	3214 (95.5%)			3025 (95.5%)	3214 (95.5%)	
GC mortality			.97				.97
Yes	90 (2.8%)	94 (2.8%)			88 (2.8%)	94 (2.8%)	
No	3147 (97.2%)	3271 (97.2%)			3080 (97.2%)	3271 (97.2%)	

[a]Among 3365 trial participants, information on age, sex, and interventions were available for all. We missed information on baseline gastric lesions for 21 participants. Information on smoking and alcohol drinking was missed for the same 112 participants. Information on dietary factors was missed for 80 participants.

[b]Subjects with missing information on the major independent variables or covariates were excluded from the multivariable models.

© 2020 Guo Y et al. *JAMA Network Open*.

eTable 2. Effects of Vitamin Supplementation on Gastric Lesion Progression Stratified by Lifestyle Factors

	Placebo (n/n)[a]	Active (n/n)[a]	OR (95%CI)[b]	*P* for interaction[c]
**Overall**	**640/1471**	**612/1440**	**0.97 (0.83-1.14)**	
Smoking				.34
Ever	295/629	292/618	1.06 (0.83-1.34)	
Never	345/842	320/822	0.91 (0.74-1.12)	
Alcohol				.94
Ever	308/660	310/682	0.97 (0.77-1.23)	
Never	332/811	302/758	0.96 (0.78-1.19)	
Grain (kg/y)[d]				.15
<225	277/611	261/631	0.83 (0.66-1.06)	
≥225	347/826	339/782	1.08 (0.87-1.33)	
Meat (kg/y)[d]				.27
<8	304/698	282/710	0.88 (0.70-1.11)	
≥8	320/739	318/703	1.06 (0.85-1.32)	
Total vegetables and fruits (kg/y)[d]				.08
<92	299/696	278/709	0.84 (0.67-1.05)	
≥92	325/741	322/704	1.12 (0.90-1.40)	
Total fresh vegetables and fruits (kg/y)[d]				.02
<81	305/702	276/703	0.80 (0.64-1.01)	
≥81	319/735	324/710	1.17 (0.94-1.46)	

[a]Number of subjects with an event divided by total number of subjects. Those with missing data on the examined independent factor or other covariates were excluded from the multivariable models.

[b]Logistic regression adjusted for baseline histology, age, sex, history of ever using alcohol, and history of ever smoking.

[c]*P* values for interactions were calculated by adding the interaction term between two items into the regression models, in addition to the indicators of the two items being analyzed.

[d]Levels correspond to median distribution of intake between two groups.

© 2020 Guo Y et al. *JAMA Network Open.*

eTable 3. Effects of Vitamin Supplementation on the Progression of Gastric Lesions, Stratified by Consumption of Vegetables and Fruit Among Participants With and Without *H pylori* Infection

	***H. pylori* positive**					***H. pylori* negative**			
	**Placebo (n/n)[a]**	**Active (n/n)[a]**	**OR (95%CI)[b]**	***P* for interaction[c]**		**Placebo (n/n)[a]**	**Active (n/n)[a]**	**OR (95%CI)[b]**	***P* for interaction[c]**
Total vegetables and fruits (kg/y)[d]				.94					.003
<92	211/483	207/475	0.98 (0.75-1.30)			88/213	71/234	0.56 (0.36-0.86)	
≥92	248/490	234/472	1.01 (0.77-1.33)			77/251	88/232	1.42 (0.95-2.13)	
Total fresh vegetables and fruits (kg/y)[d]				.68					.001
<81	216/488	204/466	0.95 (0.72-1.25)			89/214	72/237	0.53 (0.35-0.82)	
≥81	243/485	237/481	1.03 (0.79-1.36)			76/250	87/229	1.48 (0.98-2.22)	

[a]Number of subjects with an event divided by total number of subjects. Those with missing data on the examined independent factor or other covariates were excluded from the multivariable models.

[b]Logistic regression adjusted for baseline histology, age, sex, history of ever using alcohol, and history of ever smoking.

[c]*P* values for interactions were calculated by adding the interaction term between two items into the regression models, in addition to the indicators of the two items being analyzed.

[d]Levels correspond to median distribution of intake between two groups.

© 2020 Guo Y et al. *JAMA Network Open*.

eTable 4. Statistical Power for Association and Subgroup Analyses[a]

	Gastric cancer incidence[b]	Gastric cancer mortality[c]	Gastric lesion progression[b]
**Main analyses**			
Smoking	>0.99	>0.99	>0.99
Alcohol	>0.99	0.41	>0.99
Grain	0.19	0.40	0.10
Meat	0.06	>0.99	0.79
Total vegetables and fruit	0.20	0.13	0.98
Total fresh vegetables and fruit	0.09	0.52	0.91
**Subgroup analyses**			
**Vitamin supplementation**			
Overall	>0.99	>0.99	0.19
Smoking			
Ever	0.98	>0.99	0.06
Never	0.98	>0.99	0.42
Alcohol			
Ever	>0.99	>0.99	0.14
Never	0.82	>0.99	0.14
Grain (kg/y)[d]			
<225	0.87	>0.99	0.79
≥225	>0.99	>0.99	0.20
Meat (kg/y)[d]			
<8	0.98	>0.99	0.84
≥8	0.99	>0.99	0.30
Total vegetables and fruit (kg/y)[d]			
<92	>0.99	>0.99	0.82
≥92	0.86	>0.99	0.28
Total fresh vegetables and fruit (kg/y)[d]			
<81	>0.99	>0.99	0.88
≥81	0.71	>0.99	0.39
**Garlic supplementation**			
Overall	0.74	>0.99	0.10
Smoking			
Ever	0.65	>0.99	0.29
Never	0.20	>0.99	0.61
Alcohol			
Ever	0.05	0.35	0.16
Never	0.98	>0.99	0.43
Grain (kg/y)[d]			
<225	0.07	>0.99	0.14
≥225	0.93	>0.99	0.23

© 2020 Guo Y et al. *JAMA Network Open*.

Meat (kg/y) [d]			
<8	0.28	>0.99	0.68
≥8	0.45	>0.99	0.42
Total vegetables and fruit (kg/y) [d]			
<92	0.75	>0.99	0.21
≥92	0.07	>0.99	0.08
Total fresh vegetables and fruit (kg/y) [d]			
<81	0.76	>0.99	0.07
≥81	0.07	>0.99	0.05

[a]The power was calculated based on the remaining number of participants in the multivariable models after the exclusion of those missing main independent variable or covariates.

[b]Based on logistic regression and α=0.05.

[c]Based on cox regression and α=0.05.

[d]Levels correspond to median distribution of intake between two groups.

© 2020 Guo Y et al. *JAMA Network Open*.
