# Supplementary material for: Association Between Lifestyle Factors, Vitamin and Garlic Supplementation, and Gastric Cancer Outcomes: A Secondary Analysis of a Randomized Clinical Trial
Source: JAMA Netw Open. 2020 Jun 26;3(6):e206628. doi: 10.1001/jamanetworkopen.2020.6628 (PMC7320300; doi:10.1001/jamanetworkopen.2020.6628)
Supplement: Supplement 2. — Data Sharing Statement [file jamanetwopen-3-e206628-s002.pdf]

Data Sharing Statement

Guo. Association Between Lifestyle Factors, Vitamin and Garlic Supplementation, and Gastric Cancer Outcomes. *JAMA Netw* Open. Published June 26, 2020. 10.1001/jamanetworkopen.2020.6628

Data
Data available: Yes
Data types: Participant data with identifiers
How to access data: Further information on the intervention trial available upon request.
When available: With publication

Supporting Documents
Document types: Informed consent form
How to access documents: Further information on the intervention trial available upon request.
When available: With publication

Additional Information
Who can access the data: Researchers whose proposed use of the data has been approved
Types of analyses: For a specified purpose
Mechanisms of data availability: After approval of a proposal
